# The Medical Society of the State of New York

**Published:** 1889-02

**Authors:** 


### *THE MEDICAL SOCIETY OF THE STATE OF NEW YORK.*

Provisional programme of papers and discussions for the eighty-third annual meeting, in the City Hall, at Albany, on Tuesday, Wednesday, and Thursday, February 5th, 6th and 7th, 1889.

OFFICERS OF THE SOCIETY.

*President*—Samuel B. Ward, Albany; *Vice-President*—A. Walter Suiter, Herkimer; *Secretary*—William Manlius Smith, Syracuse; *Treasurer*—Charles H. Porter, Albany.

*Committee of Arrangements*—F. C. Curtis, of Albany; Edward L. Partridge, of New York, and C. S. Merrill, of Albany.

*Committee on By-Laws.*—George Seymour, of Utica; F. A. Castle, of New York; Wm. Manlius Smith, of Syracuse.

*Committee on Hygiene*—E. V. Stoddard, of Rochester; A. N. Bell, of Brooklyn; E. F. Brush, of Mt. Vernon; J. P. Creveling, of Auburn; Alfred Mercer, of Syracuse; W. H. Bailey, of Albany; W. C. Bailey, of Albion.

*Committee on Legislation*—Alexander Hadden, of New York; H. T. Pierce, of New York; F. C. Curtis, of Albany.

*Committee on Ethics*—A. Jacobi, of New York; Arthur Mathewson, of Brooklyn; H. R. Hopkins, of Buffalo.

*Committee on Prize Essays*—George F. Shrady of New York; F. P. Foster, of New York; Eugene Beach, of Gloversville.

*Committtte of Publication*—W. M. Smith, of Syracuse; C. H. Porter, of Albany; Daniel Lewis, of New York; O. B. Douglas, of New York.

FIRST DAY.

*Morning Session, 9.30 o'clock.*

President's Inaugural Address; Appointment of Committees; Executive Business.

PAPERS.—*Medical Section*—"The Municipal Control of Diphtheria," Charles Storer, Amsterdam. "Vertigo: Its Causation and Therapeutics," J. Leonard

Corning, New York. "The Prevention and Treatment of Typhoid Fever," Stephen S. Burt, New York. "Some Sanitary Suggestions," L. E. Felton, Potsdam, N. Y. "On Some of the Minor Types of Pneumonia," T. E. Satterthwaite, New York. "The Prevention of Summer Diarrhea among Infants, Viewed in the Light of the Lesions," L. Emmett Holt, New York. "Septic Poisoning in Early Life," Henry D. Chapin, N. Y. "The Treatment of Chronic Constipation by Electricity," G. M. Hammond, New York.

*Surgical Section*—"Diagnostic Value of Spinal Rotation," Bernard Bartow, Buffalo. "Recent Advances in the Treatment of Hernia," W. B. De Garmo, New York. "The Orthopedic Treatment of Tubercular Disease of the Knee in Children," V. P. Gibney, New York. "Operative Treatment of an Hyperostosis of the External Auditory Canal," T. R. Pooley, New York. "The Treatment of Acute Inflammation of the Middle Ear," Gorham Bacon, New York. "Membranous Rhinitis," Frank H. Potter, Buffalo. "The Efficacy of the Older Methods of Treating Nasal Disease Contrasted with those of To-day," Clarence C. Rice, New York. "Curative Effect of Convex Glasses in the Strabismus of Young Children," O. D. Pomeroy, New York.

*Afternoon Session, 2.30 o'clock.*

PAPERS.—"Vaginal Hysterectomy," James B. Hunter, New York. "The Importance of the Early Recognition of Cancer of the Cervix Uteri," Henry C. Coe, New York. "Report of Committee on Blindness," Lucien Howe, Buffalo; E. V. Stoddard, Rochester. "The Spectroscopic Examination of the Blood and Certain Diseases of the Eye;" "Purulent Conjunctivitis of Infants and Blindness in the State of New York," Lucien Howe, Buffalo.

*Evening Session, 7.30 o'clock.*

"Office Photography with the Flash Light," Henry G. Piffard, New York. "Cranial Measurements in Twenty Cases of Infantile Cerebral Hemiplegia," Edward D. Fisher and Frederick Peterson, New York. "Treatment of Hypertrophies at the Base of the Tongue," W. C. Phillips, New York. "A Case of Intubation of the Larynx," Wm. Hailes, Albany.

Reception by the President at his residence, 9 o'clock.

SECOND DAY.

*Morning Session, 9.30 o'clock.*

Executive Business.

PAPERS.—*Medical Section*—"The Practical Application of Acoustics to the Physical Signs of the Human Chest in Diagnosis," J. R. Leaming, New York.

"Management of Cardiac Dilatation Based on its Etiology," A. L. Loomis, New York. *Discussion*—By Drs. Westbrook, Jacobi, and Stoddard.

"The Etiology of Croupous Pneumonia," Geo. M. Sternberg, U. S. A. *Discussion*—By Drs. Loomis, Leaming, Satterthwaite, Ely, Hailes, Jacobi, and Wm. H. Porter.

"Amblyopia in Diabetes, with a Report of Six Cases," W. O. Moore, New York.

*Surgical Section*—"Kocher's Method of Reducing Sub-Coracoid Dislocation of the Humerus," Charles A. Powers, New York.

"Contribution to Surgery of the Spine," Robert Abbe, New York. *Discussion*—By Drs. Weir, Pilcher, and Gibney.

"The Management of Hip-joint Disease," A. M. Phelps, New York. *Discussion*—By Drs. Gibney, and Shaffer.

"Treatment of the So called Perityphlitic Abscesses," R. F. Weir, New York.

"Considerations Concerning the Operation for Hard Cataract," Henry D. Noyes, New York. *Discussion*—By Drs. Bull, Pooley, Gruening, Merrill, Derby, Knapp, and Webster.

"The Use of Prismatic Spectacles for the Relief of Asthenopia Caused by Insufficiency of the Ocular Muscles, with Reports of Cases," D. Webster, New York. *Discussion*—By Drs. Noyes, Gruening, Moore, Derby, Bull, and Pomeroy.

"The Treatment of Pannus," E. Gruening, New York.

*Afternoon Session, 2.30 o'clock.*

Annual Address by the President.

PAPERS.—"The Medical Treatment of Diseases of the Urinary Organs in Children," A. Jacobi, New York. "A Contribution to Surgery," L. S. Pilcher, Brooklyn.

"Chronic Headache," C. L. Dana, New York. *Discussion*—By Drs. Gray and Hopkins.

"Basedow's Disease," H. R. Hopkins, Buffalo.

*Evening Session, 7 o'clock.*

Annual Dinner of the Society.

THIRD DAY.

*Final Session.*

"A Clinical Study on Alopecia Areata and its Treatment," L. Duncan Bulkley, New York. *Discussion*—By Drs. Robinson and Fox.

"The Disorders of Menstruation," Andrew F. Currier, New York. "The Uses of Galvanism in Gynecology," W. E. Ford, Utica. "Inferior Dental Neurectomy," G. H. Glass, Utica. "Inebriety from a Medical Point of View," T. D. Crothers, Delegate from Connecticut State Medical Society.

